# Characterization of a rat model of bortezomib‐induced painful neuropathy

**DOI:** 10.1111/bph.14063

**Published:** 2017-10-29

**Authors:** Natalie A Duggett, Sarah J L Flatters

**Affiliations:** ^1^ Wolfson Centre for Age‐Related Diseases, Institute of Psychiatry, Psychology and Neuroscience King's College London London UK

## Abstract

**Background and Purpose:**

Bortezomib (Velcade®) is a breakthrough treatment for multiple myeloma, significantly improving patient survival. However, its use is limited by painful neuropathy often resulting in dose reduction/cessation of first‐line treatment due to lack of treatment. The aim of this study was to characterize a clinically relevant rat model of bortezomib‐induced painful neuropathy, using established evoked measures and novel ethological techniques, to aid drug discovery.

**Experimental Approach:**

Adult male Sprague–Dawley rats were injected i.p. with 0.1 and 0.2 mg·kg^−1^ bortezomib, or its vehicle, on days 0, 3, 7 and 10. Multiple behavioural approaches were utilized: mechanical hypersensitivity, cold allodynia, heat hypersensitivity, motor co‐ordination, burrowing and voluntary wheel running. At maximal bortezomib‐induced mechanical hypersensitivity, 200 mg·kg^−1^ ethosuximide/vehicle and 100 mg·kg^−1^ phenyl N‐tert‐butylnitrone (PBN)/vehicle were administered i.p. in separate experiments, and mechanical hypersensitivity assessed 1, 3 and 24 h later.

**Key Results:**

Bortezomib induced dose‐related mechanical hypersensitivity for up to 80 days. Bortezomib induced short‐term cold allodynia, but no significant change in heat hypersensitivity, motor co‐ordination, voluntary wheel running and burrowing behaviour compared to vehicle‐treated controls. Systemic PBN and ethosuximide significantly ameliorated bortezomib‐induced mechanical hypersensitivity.

**Conclusions and Implications:**

These data characterize a reproducible rat model of clinical‐grade bortezomib‐induced neuropathy demonstrating long‐lasting pain behaviours to evoked stimuli. Inhibition by ethosuximide and PBN suggests involvement of calcium and/or ROS in bortezomib‐induced painful neuropathy. These drugs could be used as preclinical positive controls to assess novel analgesics. As ethosuximide is widely used clinically, translation to the clinic to treat bortezomib‐induced painful neuropathy may be possible.

**Abbreviations**
ATF3: activating transcription factor 3
BIPN: bortezomib‐induced painful neuropathy
DRG: dorsal root ganglia
IENF: intraepidermal nerve fibre
PBN: phenyl‐N‐tert‐butylnitrone

## Introduction

Bortezomib (Velcade®) is the first of a new family of chemotherapeutics known as proteasome inhibitors. Primarily, bortezomib is a first‐line agent in treatment of newly diagnosed and relapsed multiple myeloma (Engelhardt *et al.,* 2010), with further trials showing effectiveness against non‐Hodgkin's lymphoma (Bose *et al.,* 2014) and solid carcinomas, including renal, prostate and ovarian tumours (Cusack, 2003; Huang *et al.,* 2014). Bortezomib is a boronic acid dipeptide which reversibly binds and inhibits the 20S catalytic subunit of the 26S proteasome (Adams, 2002). Proteasome inhibition has been shown to interrupt critical cell pathways, including the ubiquitin‐proteasome pathway and p53, JNK and NFκB activity, to induce apoptosis (Hideshima *et al.,* 2003; Voorhees *et al.,* 2003). Malignant cells are more sensitive to proteasome inhibition, undergoing apoptosis more readily than healthy cells (reviewed in Voorhees *et al.,* 2003). Bortezomib can indirectly polymerise tubulin, causing microtubule stabilization, axonal transport inhibition and G_2_M phase cell cycle arrest (Poruchynsky *et al.,* 2008; Rapino *et al.,* 2013; Staff *et al.,* 2013).

Multiple studies report a high incidence (36–75%) of peripheral neuropathy following bortezomib administration (reviewed in Argyriou *et al.,* 2008; Badros *et al.,* 2007; Cavaletti and Jakubowiak, 2010; Seretny *et al.,* 2014). The high incidence of neuropathy occurs across patient groups, whether newly diagnosed or relapsed/progression multiple myeloma (52 vs. 55%, respectively; Corso *et al.,* 2010). Previously untreated patients, who received bortezomib, have shown less frequent neuropathic pain which rapidly improved, compared to relapsed patients treated with bortezomib. However, sensory symptoms, including paraesthesia and dysesthesia, persisted significantly longer in previously untreated patients (Corso *et al.,* 2010). Multicentre phase 2 and phase 3 studies have shown that up to 75% of patients with bortezomib‐induced painful neuropathy (BIPN) had resolution of symptoms within 2–3 months of treatment cessation or dose‐modification (reviewed in Delforge *et al.,* 2010). However, 25% of BIPN patients remain unaffected by dose alteration and have their quality of life adversely affected for long periods (13–20 months) following treatment (13–20 months; Corso *et al.,* 2010; Richardson *et al.,* 2006; Richardson *et al.,* 2009). BIPN is characterized by painful paraesthesia, hypoesthesia, cold allodynia and heat hypersensitivity with little motor impairment reported (Cata *et al.,* 2007; Argyriou *et al.,* 2008; Cavaletti and Jakubowiak, 2010). Currently, there are no effective treatments for bortezomib‐induced peripheral neuropathy. At present, dose‐reduction and change in dosing regimen/administration is recommended to limit establishment of BIPN (Moreau *et al.,* 2011; 2012; Argyriou *et al.,* 2014).

A number of animal models of BIPN exist in both mice and rats, recently reviewed by Hopkins *et al*. (2016). In this study, Velcade® was administered at regular intervals to mimic the first bortezomib treatment cycle. The first aim of this investigation was to comprehensively characterize a rat model of BIPN using several behavioural techniques, encompassing both established measures to evoked pain stimuli and novel ethological activity measures. These techniques were used to investigate patient‐reported symptoms, whilst using clinically relevant dosage, cycle regimen and grade of bortezomib. The second aim of this study was to assess if novel analgesics shown to be effective in a rat model of paclitaxel‐induced painful neuropathy were also effective in this model. This was done to identify potential positive controls and mechanistic insight for the assessment of novel‐analgesics against BIPN.

## Methods

### Animals

Adult male Sprague–Dawley rats (180–200 g; Envigo) were housed in cages of four to five, in a climate‐controlled environment with a 12 h light/dark cycle. Animals had free access to food and water, and all cages contained sawdust and environmental enrichment materials. Animal studies are reported in compliance with the ARRIVE guidelines (Kilkenny *et al*., 2010; McGrath and Lilley, 2015). All procedures were conducted in strict accordance with the UK Animals (Scientific Procedures) Act, 1986, and the IASP ethical guidelines (Zimmerman, 1983). Protocols used were approved by the Ethics Review Panel of King's College London and conducted under the UK Home Office project licence 70/8015.

### Drug administration

Velcade®, clinical grade bortezomib (Millennium Pharmaceuticals Inc., Takeda Pharmaceutical Company Limited), was used to induce bortezomib‐induced peripheral neuropathy. Powdered Velcade® was reconstituted with 0.9% sterile saline (Fresenius Kabi, UK) to achieve a solution with a final concentration of 0.2 mg·mL^−1^. A vehicle solution of 0.2 mg·mL^−1^ D‐mannitol (Sigma, UK), in 0.9% sterile saline, was used to replicate clinical Velcade®. Animals received 0.1 or 0.2 mg·kg^−1^ bortezomib i.p. or an equal volume of vehicle solution on days 0, 3, 7 and 10. Dosage and administration schedule was based on clinical use of Velcade® [1.3 mg·m^−2^ (Conversion factor: Nair and Jacob, 2016) on days 0, 3, 7 and 10 (Moreau *et al.,* 2012)]. Velcade® has been shown to induce dose‐dependent proteasome inhibition, with a single dose of 1.38 mg·m^−2^ yielding 74 ± 2% inhibition, which returned towards baseline after 72 h (More detail: Orlowski *et al.,* 2002).

### Mechanical hypersensitivity

As described previously (Griffiths and Flatters, 2015; Duggett *et al.,* 2016), animals were habituated to the testing environment two to three times on separate days prior to assessment of baseline mechanical responses. The testing environment consisted of an elevated Perspex box with a wire‐rung floor, with clear dividers creating 15 × 16 × 21 cm boxes for each animal. Withdrawal responses to von Frey filaments of 4, 8 and 15 g bending force (Touch‐Test™ Sensory Evaluators, Linton Instrumentation, UK) were used to assess mechanical hypersensitivity as described previously (Griffiths and Flatters, 2015), with scores from both hind paws added together to give a maximum possible score of 10 for each bending force filament weight. In brief, each filament was applied five times, for 5 s, to the midplantar region of the hind paw. All animals were tested using one von Frey filament on one hind paw before beginning to test the other hind paw with the same bending force filament. Three baseline measurements, taken on separate days, were recorded prior to administration of bortezomib/vehicle, and von Frey withdrawal responses to all bending forces were then measured at 1–2 week intervals in order to fully characterize the time course of bortezomib‐induced mechanical hypersensitivity, *n* = 7 per group. Mechanical hypersensitivity was assessed between 08:00 and 11:00 h on all occasions.

### Cold allodynia

The testing environment for cold hypersensitivity testing was the same as that described above for mechanical hypersensitivity and was assessed as detailed previously (Fidanboylu *et al.,* 2011). In brief, 50 μL of acetone (Sigma, UK) was applied to the plantar surface of each hind paw and a stopwatch started. Responses were scored as follows: 0, no response; 1, a single response (lifting, flicking or stamping the paw); 2, multiple/prolonged responses; 3, repeated responses and licking of the plantar surface. Responses were graded initially over the first 20 s following acetone application. A score of zero was given if the animal did not respond in the first 20 s following application. If the animal did respond within this time frame, the animal's response was assessed for an additional 20 s. Each hind paw was tested three times, giving a total score out of 18 for each animal. Hind paw testing was non‐consecutive, with each animal tested on one hind paw before returning to the original animal to test the same hind paw again, *n* = 7 per group. The minimum time between acetone applications to the same hind paw was 8 min. Cold allodynia was tested on days 0, 6, 11, 14, 18, 21, 26, 33, 42, 48, 54, 61 and 74.

### Thermal hyperalgesia

Heat hyperalgesia was assessed using an infrared heat source (Ugo‐Basile, Italy). An infrared beam was targeted to the plantar heel surface of each hind paw, which automatically cuts off when paw was withdrawn. The latency from initiation of stimulation to paw withdrawal was displayed and recorded. Withdrawal latency was assessed for each hind paw in turn, three times in a non‐consecutive manner to avoid sensitization. For example, each animal had their left hind paw tested once, before the first animal was tested a second time on the left paw. The average time between heat stimulation to the same hind paw was 6 min. Latencies for each hind paw were added together and averaged to give the mean of all six measurements, *n* = 8 per group. The infrared intensity was maintained at 150–160 mW·cm^−3^ as measured using a Heat Flux Radiometer (Ugo‐Basile, Italy). Thermal hyperalgesia was assessed on days 0, 5, 11, 14, 18, 21, 28, 35, 46 and 63.

### Motor co‐ordination

Motor performance was assessed using an accelerating Rota‐rod (Ugo‐Basile, Italy). Animals were positioned on the rota‐rod at an initial speed of 4 rpm, with speed increasing 40 rpm over 300 s; the latency for the rat to fall onto the sensor platform was recorded. Animals were habituated to the rota‐rod on three separate occasions and were required to sustain movement on the rota‐rod for a minimum of 60 s on two of these occasions in order to be included in the study. Two baseline assessments were conducted for each animal on different days, with one trial conducted per time point assessed, *n* = 8 per group. Motor coordination was assessed on days 0, 5, 11, 19, 26, 33, 46 and 60.

### Burrowing behaviour

Burrowing behaviour was assessed using custom‐made burrowing tubes in a protocol based on work previously described (Deacon, 2006; Andrews *et al.,* 2012; Huang *et al.,* 2013). Burrowing tubes were 32 cm in length, 10 cm in diameter, and had one end sealed. The open end was raised by approximately 6 cm. Animals were housed in pairs and habituated to empty burrowing tubes in their home cage for a minimum of 24 h. Following this, training was initiated. Training sessions consisted of the cage mates being moved to a fresh home‐cage which contained the burrowing tube, which they had been habituated to, filled with 2500 g of 5 mm shingle (Porton Garden, Aquatics and Pets Centre, UK) and a disposable inco pad (Guy's Hospital) in lieu of sawdust. Animals were trained in pairs for 2 h, from 16:00 to 18:00 h, after which they were returned to their original home cage with sawdust and environmental enrichment materials, without the burrowing tube. This was repeated on two consecutive days, if any pairs of animals did not burrow in the first training session the pairings were swapped with animals that did show burrowing activity. Animals had free access to food and water at all times.

Following training, a baseline burrowing measurement was taken the following day. Animals were individually housed with a burrowing tube containing 2500 g 5 mm shingle and an inco pad from 16:00 to 18:00 h. After this time, animals were returned to their home‐cage and respective cage mate. The quantity of gravel dislodged and latency to start burrowing were recorded, and the weight of gravel dislodged was used to group‐match the animals prior to drug administration, to ensure similar baseline burrowing behaviour between treatment groups, *n* = 6 vehicle, *n* = 7 bortezomib. Animals were individually placed in the burrowing set‐up from 16:00 to 18:00 h on day 4, day 19 and day 33 post‐initial bortezomib/vehicle administration with latency and quantity of gravel displaced recorded at each time point. Other than during the training period, animals were exposed to the same burrow and gravel and were returned to the same home cage and cage mate for the duration of the study.

### Voluntary wheel running

Spontaneous activity was assessed using Activity Wheels, model, BIO‐ACTIVW‐R (Bioseb, Boulogne, France). This software allowed recording of activity within a cage similar to that of the animal's home cage, with overall dimensions of 48 × 31.5 × 47 cm and wheel dimension of 34 cm height, 7 cm width. Cages were Type III polycarbonate, and wheels were made of stainless steel. Animals had access to food and water *ad libitum* and free access to the wheel. Activity wheel cages contained sawdust only and were kept within the room that the rats were normally housed. Animals were individually housed in activity wheel cages during recording periods, after which they were returned to their home cage and cage mates. The activity wheels were connected to a computer and ActivWheel software which automatically recorded a number of parameters: active time, distance travelled, speed, acceleration and access count, throughout the recording period. Animals were habituated to the activity wheel cage for 1 h during the day prior to overnight recordings. As rodents are nocturnal and previous studies have shown that the majority of wheel running occurs during the dark cycle (Eikelboom and Mills, 1988), this period was focussed upon during this investigation. For baseline recordings (day 0), animals were placed in the activity wheel cage at 18:00 h, until 09:00 h the following day. Animals were split into two groups based on the total distances travelled at baseline recorded during the dark cycle. Animals were placed in their activity wheel cage again on day 4, day 19 and day 33, for 1 h during the light cycle to habituate and then from 18:00 to 09:00 h the following day. Data graphed in Figure 7 were obtained during the dark cycle, 19:30 to 06:30 h (avoiding the dusk/dawn phase). Animals were always exposed to the same activity wheel cage and returned to the same home cage and cage mates, *n* = 12 per group.

### Histological analyses

In order to investigate whether bortezomib administration induced neuronal damage, several parameters were investigated. Axon myelination was investigated using osmium staining conducted by Carl Hobbs; at day 24, sciatic nerve samples were taken from bortezomib and vehicle‐treated animals. Samples were incubated for 24 h in 10% formalin, before being washed with distilled water three times, and incubation with 0.1% osmium for 2 h at room temperature (RT). Tissue specimens were washed with distilled water for 15 min three times, before being embedded in paraffin wax. Cross‐sectional nerve sections were cut at 8 μm, mounted and air‐dried. Slides were incubated at 62°C for 2 h before dehydrating with xylene for 10 min and coverslipping with DPX. Slides were randomized and regions selected to count axons highlighted by S.J.L.F. Axons that did not have a clear lumen or suggested compromised myelin were not included in the assessment. The experimenter was blind throughout analyses. Sample size was based on number of animals, *n* = 5 per group.

Additionally, activating transcription factor 3 (ATF3) immunofluorescence was conducted on 16 μm L5 dorsal root ganglion (DRG) sections cut onto slides from bortezomib and vehicle‐treated animals at day 24, peak mechanical hypersensitivity. Anti‐ATF3 primary antibody (1:500, O/N at 4°C, Santa‐Cruz Biotechnology, USA) was used, followed by goat anti‐rabbit CY3 secondary antibody (1:400, 90 min at RT, Jackson ImmunoResearch Laboratories Inc., USA) using the methodology previously described (Flatters, 2008). Non‐sequential sections were analysed with total cell counts taken from three sections per animal. Sample size was based on number of animals, (*n* = 6 per group).

Intraepidermal nerve fibres (IENFs) were analysed in the hind paw skin of bortezomib and vehicle‐treated animals at day 24. Hind paw skin was sectioned onto slides at 10 μM and hydrated for 10 min in PBS. Sections were then blocked for 1 h at RT in a diluent solution (1% BSA, 0.1% Triton in PBS). Slides were incubated with pan‐neuronal marker protein gene product 9.5 (anti‐PGP 9.5, 1:5000, Ultraclone Ltd, UK) overnight in diluent solution at RT. Slides were then washed three times for 10 min in PBS before incubation with goat anti‐rabbit Cy3 (1:500, Jackson ImmunoResearch Laboratories Inc., USA) in diluent for 90 min at RT. Slides were washed three times for 10 min in PBS, before being coverslipped using Vectashield with DAPI to identify the dermal‐epidermal border. Scoring criteria were based on guidelines recommended by the European Federation of Neurological Societies (Lauria *et al.,* 2005). For each animal, 32 images of skin IENFs were analysed. Images were taken from four fields of view per skin section, with eight non‐sequential sections analysed per animal under blind conditions. Sample size was based on number of animals (*n* = 6 per group).

### Drug treatment studies on established bortezomib‐induced mechanical hypersensitivity

On day 21 post initial injection of bortezomib, mechanical hypersensitivity to von Frey filaments was assessed (*n* = 14) as detailed above. Animals were split into two groups that displayed similar levels of mechanical hypersensitivity to 4, 8 and 15 g von Frey stimulation. Animals received 200 mg·kg^−1^ ethosuximide (dissolved in saline, Sigma, UK, *n* = 7) or saline (*n* = 7), administered i.p. All animal's mechanical hypersensitivity was then tested 1, 3 and 24 h after ethosuximide/vehicle administration.

At day 24 post initial administration of bortezomib, mechanical hypersensitivity to 4, 8 and 15 g von Frey filaments was assessed. Animals were split into matched groups based on their responses to von Frey stimulation. A total of 100 mg·kg^−1^ of reactive oxygen scavenger, phenyl‐N‐tert‐butylnitrone (PBN), dissolved in saline (Sigma, UK; *n* = 7) or saline (*n* = 7) were administered i.p. Mechanical hypersensitivity was assessed 1, 3 and 24 h after PBN/vehicle administration. Dosing for ethosuximide and PBN was based on previous studies (Flatters and Bennett, 2004; Fidanboylu *et al.,* 2011).

### Blinding and randomization

All behavioural testing was carried out by a single experimenter (N.A.D.), who was blind to the treatment group. For drug (PBN and ethosuximide) studies on established mechanical hypersensitivity, randomization of animals and all injections were performed by a different experimenter (S.J.L.F.). Animals were allocated to experimental groups based on their baseline responses to the respective behavioural test. In order to reduce the effect of extraneous variables on behavioural assessment, concurrent vehicle‐treated groups were present throughout all experiments. All bortezomib/vehicle injections were administered in the early afternoon 13:00 to 15:00 h, and ethosuximide/PBN/vehicle injections were administered between 11:00 and 12:00 h. All behaviour and drug administrations were conducted in designated procedure facilities within the King's College London Biological Services Unit. Behavioural testing was conducted in the morning, between 08:00 and11:00 h.

### Statistical analysis

All statistical analyses were carried out on raw data using GraphPad Prism 6 or IBM SPSS Statistics 23. To monitor weight gain in vehicle‐treated compared to bortezomib‐treated rats, a two‐way repeated measures ANOVA with Holm‐Sidak *post hoc* test was conducted. Mechanical hypersensitivity time course data were analysed using a two‐way ANOVA with Holm‐Sidak *post hoc* test. The time course of bortezomib‐induced cold hypersensitivity was analysed using Friedman test with Dunn's *post hoc* compared to baseline scores in each group. Heat hypersensitivity and motor coordination, in conjunction with bortezomib administration, were analysed using repeated measures two‐way ANOVA with Holm‐Sidak *post hoc* test. Spontaneous burrowing activity in bortezomib‐ and vehicle‐treated animals was analysed using repeated measures two‐way ANOVA, and one animal was removed from the vehicle group as it failed to burrow at day 33. Voluntary wheel running behaviour in conjunction with bortezomib/vehicle administration was analysed using repeated measures two‐way ANCOVA, using baseline values as the covariate. Pairwise analysis was also conducted in SPSS, with Bonferroni adjustment. The effect of ethosuximide and PBN on established bortezomib‐induced mechanical hypersensitivity was analysed using unpaired two‐tailed *t*‐tests, with pre‐ and post bortezomib data compared using paired one‐tailed *t*‐tests. Statistical significance was accepted at *P* < 0.05, with no further distinction made for *P* < 0.01. The data and statistical analysis comply with the recommendations on experimental design and analysis in pharmacology (Curtis *et al*., 2015).

### Nomenclature of targets and ligands

Key ligands in this article are hyperlinked to corresponding entries in http://www.guidetopharmacology.org, the common portal for data from the IUPHAR/BPS Guide to PHARMACOLOGY (Southan *et al.,* 2016).

## Results

**Figure 1:**
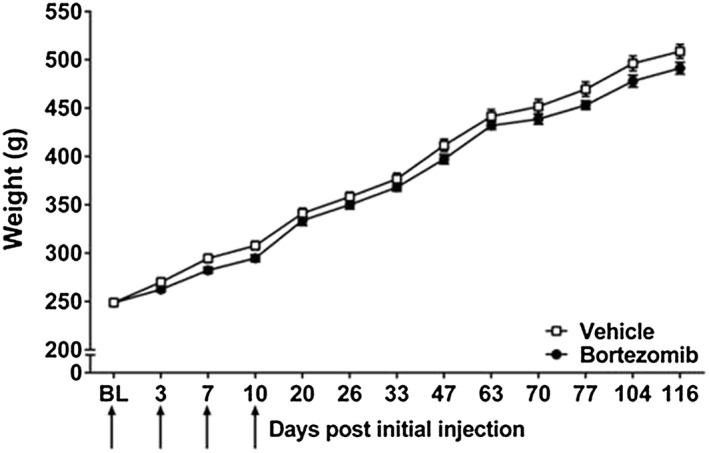
Weight gain in bortezomib‐treated and vehicle‐treated animals. Graph shows the mean ± SEM weight in g of rats, up to day 116, exposed to bortezomib or its vehicle. Arrows indicate four injections of 0.2 mg·kg^−1^ bortezomib or equivalent volume of vehicle on days 0, 3, 7 and 10. *n* = 8 per group.

All rats appeared healthy throughout the studies. Bortezomib‐ and vehicle‐treated animals gained weight throughout the investigation, and there was no significant difference between the groups throughout the time course of the study (Figure 1, *n* = 8, repeated measures two‐way ANOVA with Holm‐Sidak *post hoc* test). Bortezomib‐treated animals showed no signed of alopecia or diarrhoea, using either dosing regimen.

**Figure 2:**
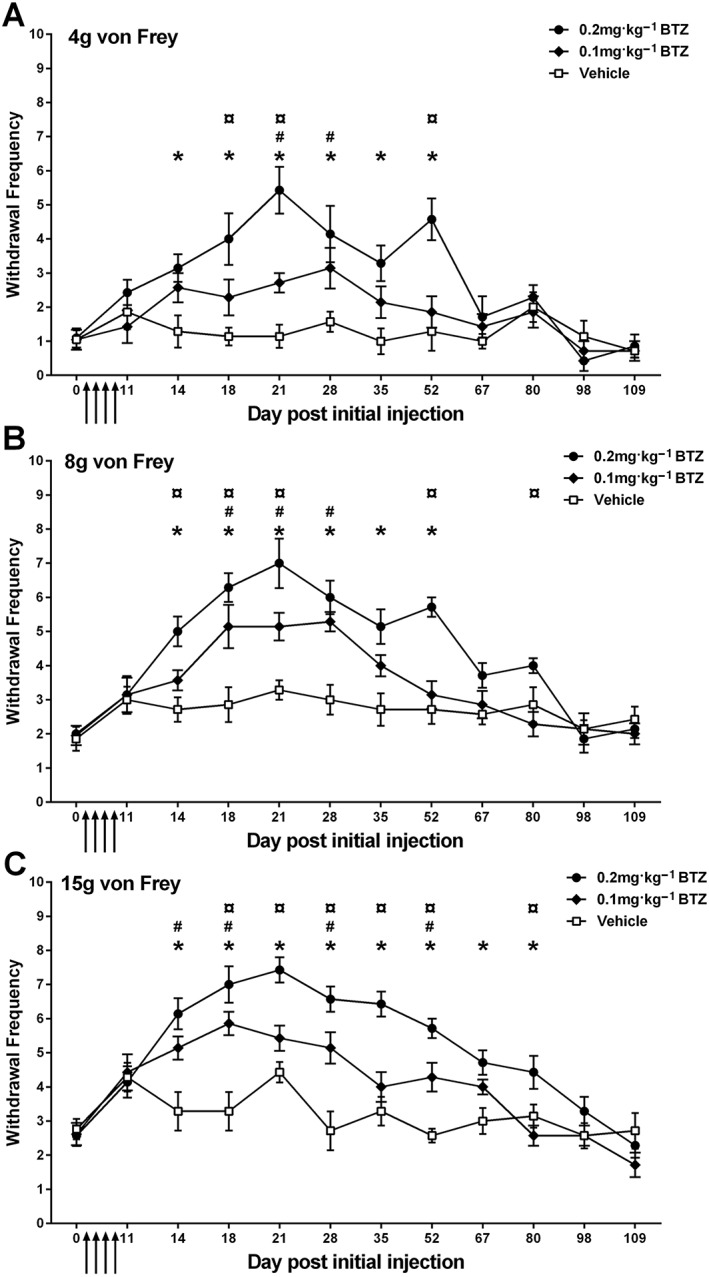
Time course of mechanical hypersensitivity induced by bortezomib (BTZ). (A–C) The mean ± SEM of the number of withdrawal responses to 4, 8 and 15 g von Frey, respectively, throughout the time course. Arrows indicate four injections of 0.1 or 0.2 mg·kg^−1^ bortezomib or equivalent volume of vehicle solution on days 0, 3, 7 and 10. *P < 0.05, vehicle versus 0.2 mg·kg^−1^ bortezomib; #*P* < 0.05, vehicle versus 0.1 mg·kg^−1^ bortezomib; ¤*P* < 0.05, 0.1 mg·kg^−1^ versus 0.2 mg·kg^−1^ bortezomib. *n* = 7 per group.

Figure 2 shows that administration of bortezomib resulted in a significant and long‐lasting mechanical hypersensitivity. Following four systemic injections of 0.1 or 0.2 mg·kg^−1^ bortezomib (cumulative dosing of 0.4 and 0.8 mg·kg^−1^, respectively) on days 0, 3, 7 and 10, there was a short delay before the onset of mechanical hypersensitivity. On day 14, a clear and significant increase in mechanical hypersensitivity was evident following both dosing regimens. A dose‐related difference in response duration was evident following intermittent bortezomib administration; 0.1 mg·kg^−1^ bortezomib‐induced mechanical hypersensitivity lasted between 28 (4 and 8 g von Frey) and 52 days (15 g von Frey), whereas 0.2 mg·kg^−1^ bortezomib induced mechanical hypersensitivity that lasted between 52 (4 and 8 g von Frey) and 80 days (15 g von Frey), when compared to concurrent vehicle‐treated controls. The mechanical hypersensitivity to bortezomib also showed significant dose‐dependent effects with 0.2 mg·kg^−1^ resulting in significantly greater mechanical hypersensitivity than dosing at 0.1 mg·kg^−1^. This was evident at days 18, 21 and 52 when stimulating at 4 g, at days 14–21, 52 and 80 when stimulating at 8 g, and at days 18–52 and 80 when stimulating at von Frey 15 g. Mechanical hypersensitivity was deemed to have fully resolved after responses returned to individual baseline responses on two separate occasions (approximately 110 days after initial bortezomib administration; *n* = 7 per group, two‐way repeated measures ANOVA with Holm‐Sidak *post hoc* test). We have reproduced this time course of mechanical hypersensitivity in three separate cohorts over the course of a year (*n* = 12 per condition, per cohort).

**Figure 3:**
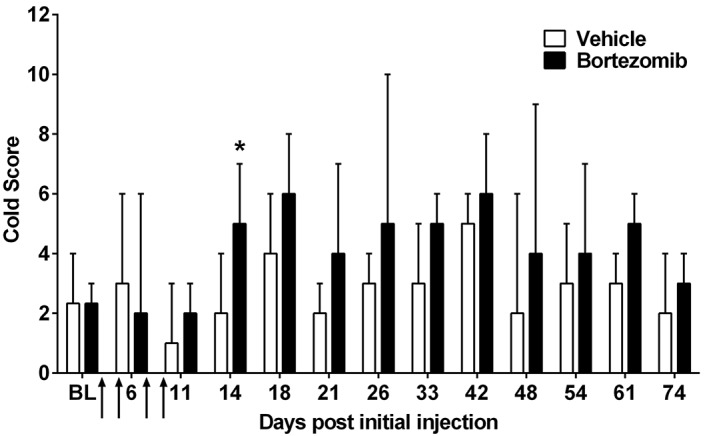
Time course of bortezomib‐induced cold hypersensitivity. Graph shows the median ± interquartile range of the animal's response to acetone application throughout the time course of bortezomib‐induced mechanical hypersensitivity. Cold hypersensitivity was evident at day 14 compared to baseline. Arrows indicate four injections of 0.2 mg·kg^−1^ bortezomib or equivalent volume of vehicle on days 0, 3, 7 and 10. *P < 0.05, *n* = 7 per group. NB. As this analysis is non‐parametric, error bars appear larger than expected from parametric analysis.

Bortezomib (dosing at 0.2 mg·kg^−1^) was shown to induce a significant cold hypersensitivity at day 14, when compared to baseline responses (see Figure 3). Vehicle‐treated animals showed no significant change in cold hypersensitivity at any time point investigated (Friedman test, Dunn's *post hoc* test comparing to baseline scores, *n* = 7). Cold hypersensitivity was monitored until concurrent mechanical hypersensitivity was deemed to have resolved. From these data, late‐phase cold hypersensitivity was not observed.

**Figure 4:**
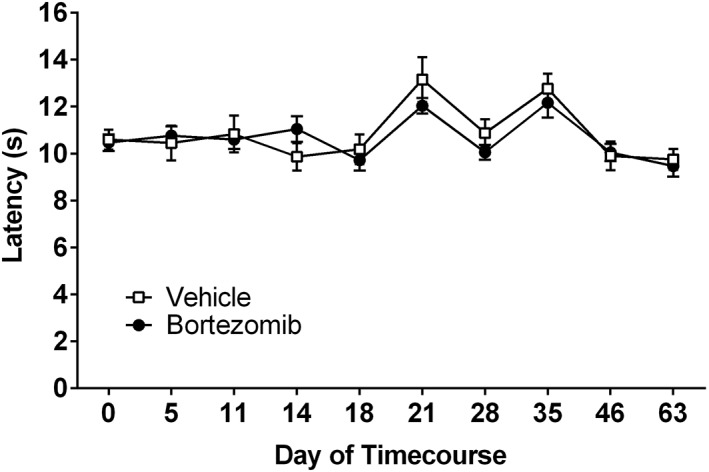
Effect of bortezomib administration on heat hypersensitivity. Graph shows the mean ± SEM of the latency in seconds to withdraw from a radiant heat stimulus. Arrows indicate four injections of 0.2 mg·kg^−1^ bortezomib or equivalent volume of vehicle, on days 0, 3, 7 and 10, *n* = 8 per group.

No significant difference was observed in the withdrawal latency to heat stimulation in bortezomib (0.2 mg·kg^−1^ dosing) and vehicle‐treated animals at any time point investigated (Figure 4, repeated measures two‐way ANOVA with Holm‐Sidak *post hoc* test, *n* = 8 per group). Points of elevated withdrawal threshold, observed at days 21 and 35 in both groups, were due to air conditioning in the testing room lowering the ambient temperature and cooling of the glass testing platform. Withdrawal latency was assessed up to day 63 following initial bortezomib/vehicle administration, following the time course during which of mechanical hypersensitivity emerges, no late‐phase heat hypersensitivity was observed.

**Figure 5:**
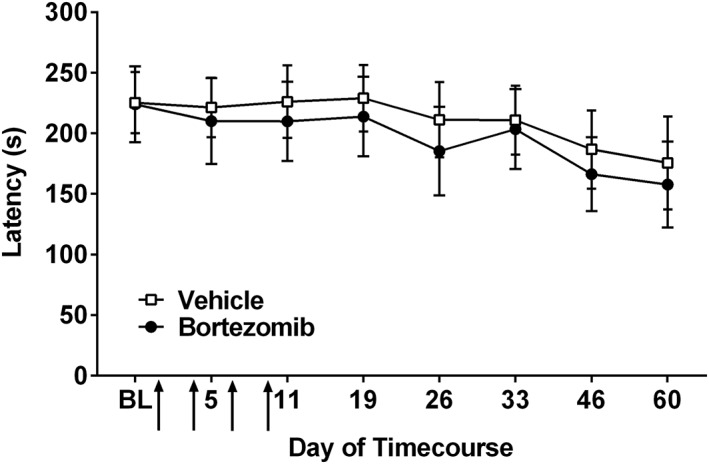
Effect of bortezomib administration on motor coordination. The graph shows the mean ± SEM of the latency in seconds, for vehicle‐ and bortezomib‐treated rats, to fall from an accelerating rota‐rod. Arrows indicate four injections of 0.2 mg·kg^−1^ bortezomib or equivalent volume of vehicle on days 0, 3, 7 and 10, *n* = 8 per group.

Administration of four doses of 0.2 mg·kg^−1^ bortezomib on days 0, 3, 7 and 10 had no significant effect on motor coordination, assessed by the animal's latency to fall from the rota‐rod, when compared to the latency of vehicle‐treated control animals (Figure 5, repeated measures two‐way ANOVA with Holm‐Sidak *post hoc* test, *n* = 8 per group). Motor coordination was assessed up to day 60 following initial bortezomib/vehicle administration, following the time course during which of mechanical hypersensitivity becomes established.

**Figure 6:**
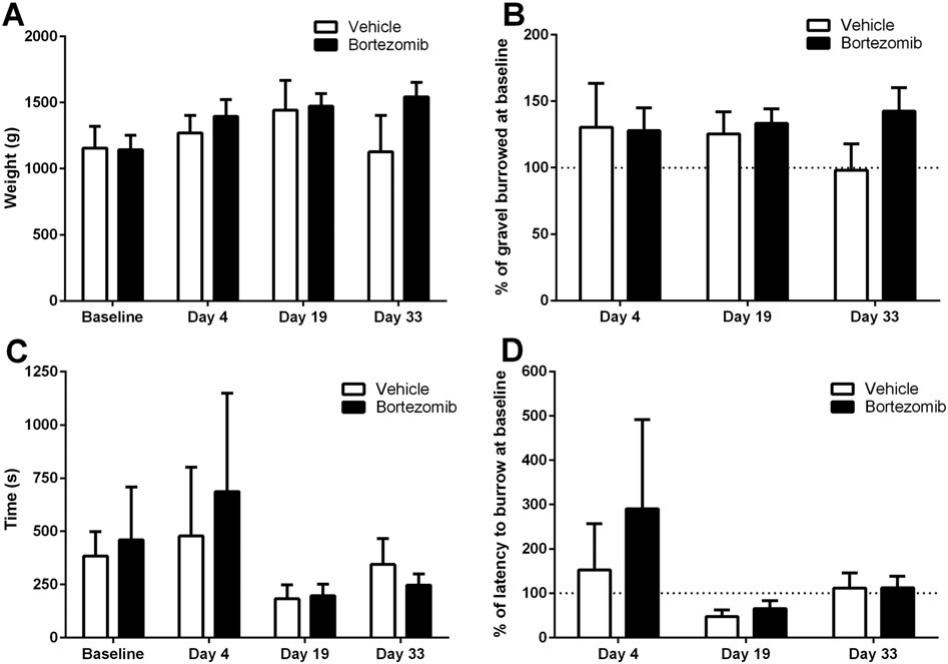
Spontaneous burrowing activity in bortezomib‐ and vehicle‐treated animals. (A) The mean ± SEM of the weight of gravel displaced at baseline, day 4, day 19 and day 33 in the time course of bortezomib‐induced mechanical hypersensitivity. (B) The gravel displaced by an animal at day 4, 19 and 33 as a percentage of their respective weight of gravel displaced at baseline. (C) The mean ± SEM latency time to burrow in seconds at baseline, day 4, day 19 and day 33. (D) The animal's latency to initiate burrowing at day 4, 19 and 33 as a percentage of their respective latency to burrow at baseline. *n* = 6 vehicle, *n* = 7 bortezomib.

As shown in Figure 6, intermittent administration of bortezomib (0.2 mg·kg^−1^ dosing) had no significant effect on burrowing behaviour when compared to that of vehicle‐treated animals at any time point investigated. Burrowing behaviour, amount of gravel displaced and latency to burrow were investigated at day 4, day 19 and day 33 post initial systemic administration of bortezomib/vehicle solution (repeated measures two‐way ANOVA with Holm‐Sidak *post hoc* test, *n* = 6 for vehicle, *n* = 7 for bortezomib). One animal was removed from the vehicle group as it did not burrow during the 2 h time period at day 33.

**Figure 7:**
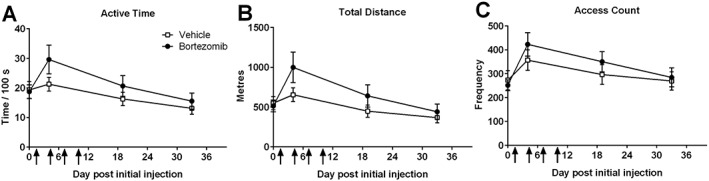
Effect of bortezomib administration on voluntary wheel running. (A) The active time animals spent in the wheel at baseline, day 4, day 20 and day 34 in the time course of bortezomib‐induced mechanical hypersensitivity. (B) The total distance animals ran in the wheel at day 4, day 20 and day 34. (C) The access count of the wheel at day 4, day 20 and day 34. Results are presented as mean± SEM; *n* = 12 per group.

Voluntary wheel running, a novel ethological behavioural test, was investigated in this study. Reported to be a natural, elective behaviour and a stress‐reduced method of testing, we also found it to be a highly variable approach. ANCOVA analysis identified that activity at baseline was a significant factor on data at later time points for all parameters measured: total distance, active time, access count, mean speed, maximal speed and maximal acceleration. The significance of an individual animal's baseline activity was evident, regardless of drug administration group. Therefore, two independent cohorts of animals were exposed to this novel behavioural approach and the data combined. Intermittent administration of bortezomib (0.2 mg·kg^−1^ dosing) was shown to have a significant effect on voluntary wheel running behaviour. Figure 7 shows that animals which received bortezomib ran greater total distances, compared to vehicle‐treated controls (repeated measures –two‐way ANCOVA with baseline values, used as the covariate, *n* = 12 per group, two separate cohorts *n* = 6 for both repeats). Although ANCOVA analysis identified that bortezomib‐treated animals ran significantly greater distances compared to vehicle‐treated animals, pairwise analysis at individual time points did not find any significant change in total distance run by bortezomib‐treated compared to vehicle‐treated animals. Bortezomib‐treated animals also showed a trend towards increased active time in the voluntary wheels, compared to vehicle‐treated controls (*P* < 0.1, repeated measures – two‐way ANCOVA with baseline values, used as the covariate, *n* = 12 per group, two separate cohorts *n* = 6 for both repeats). Although ANCOVA analysis identified that bortezomib‐treated animals ran significantly greater distances compared to vehicle‐treated animals, pairwise analysis at individual time points did not find any significant change in total distance run by bortezomib‐treated compared to vehicle‐treated animals. All pairwise analyses were non‐significant, and these analyses indicate the importance of accounting for individual animal's baseline activity in this novel assay.

**Figure 8:**
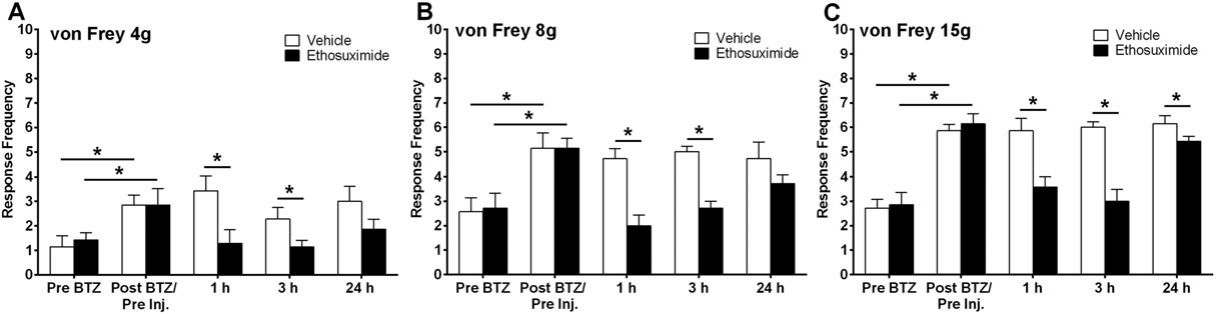
Effect of ethosuximide on established bortezomib‐induced mechanical hypersensitivity. (A–C) The effect of systemic 200 mg·kg^−1^ ethosuximide or vehicle administration on behavioural responses to von Frey 4, 8 and 15 g stimulation, respectively, on day 21 following bortezomib administration. Graphs show the mean ± SEM of response frequency to mechanical stimulation pre‐bortezomib administration (Pre bortezomib), after bortezomib but before ethosuximide/vehicle injection (Post bortezomib/Pre inj.), and then 1, 3 and 24 h following ethosuximide/vehicle administration. *P < 0.05; *n*  =  7 per group.

The effect of a single systemic administration of ethosuximide on bortezomib‐induced mechanical hypersensitivity is illustrated in Figure 8A–C. Significant mechanical hypersensitivity was induced by day 21 following administration of 0.2 mg·kg^−1^ bortezomib on days 0, 3, 7 and 10 (pre‐bortezomib vs. post‐bortezomib, *P < 0.05, paired one‐tailed *t*‐tests) at all von Frey weights tested, as expected (see Figure 2). One and 3 h post administration of 200 mg·kg^−1^ ethosuximide bortezomib‐induced mechanical hypersensitivity was markedly inhibited to all von Frey stimulation (Figure 8A–C, *P < 0.05, unpaired two‐tailed *t*‐tests). The degree of mechanical hypersensitivity inhibition by ethosuximide ranged from 41 to 60% across 4, 8 and 15 g von Frey stimulation over 1 and 3 h time points. Twenty four hours after administration, no significant effect of ethosuximide treatment was observed at 4 and 8 g von Frey filaments when compared to vehicle‐administrated bortezomib‐treated animals. However, at the 24 h time point a small, but statistically significant, analgesic effect of ethosuximide administration was still evident at the 15 g von Frey stimulation (Figure 8C, *P < 0.05, unpaired two‐tailed *t*‐tests).

**Figure 9:**
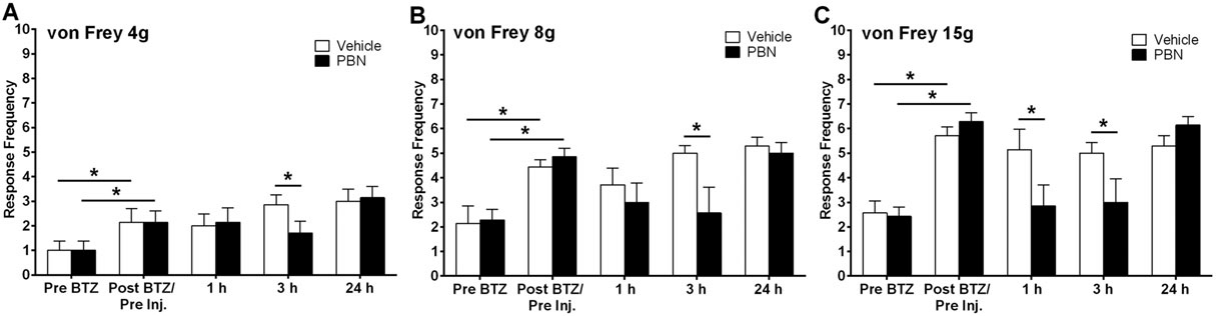
Effect of PBN on established bortezomib‐induced mechanical hypersensitivity. (A–C) The effect of systemic 100 mg·kg^−1^ PBN or vehicle administration on behavioural responses to von Frey 4, 8 and 15 g stimulation, respectively, on day 24 following bortezomib administration. Graphs show the mean ± SEM of response frequency to mechanical stimulation pre‐bortezomib administration (Pre bortezomib), after bortezomib but before PBN/vehicle injection (Post bortezomib/Pre inj.), and then 1, 3 and 24 h following PBN/vehicle administration. *P < 0.05, *n*  =  7 per group.

Figure 9A–C shows the effect of a single systemic dose of the ROS scavenger, PBN, on established bortezomib‐induced mechanical hypersensitivity. Significant mechanical hypersensitivity was apparent at day 24 following administration of 0.2 mg·kg^−1^ bortezomib on days 0, 3 7 and 10 (Pre‐bortezomib vs. post‐bortezomib, *P < 0.05, paired one‐tailed *t*‐tests) with all von Freys examined. At 1 h following 100 mg·kg^−1^ PBN administration, bortezomib‐induced mechanical hypersensitivity was inhibited at 15 g von Frey filament testing only (Figure 9C,*P < 0.05 unpaired two‐tailed *t*‐test). However, 3 h after PBN administration, bortezomib‐induced hypersensitivity was significantly reduced at all von Frey weights tested, ranging from 20 to 52% inhibition (Figure 9A–C, *P < 0.05, unpaired two‐tailed *t*‐tests). No effect of PBN on bortezomib‐induced mechanical hypersensitivity was observed 24 h after administration of PBN at any von Frey stimulation.

We looked for evidence of neurodegeneration in several ways. At peak mechanical hypersensitivity, day 24, axon myelination, ATF3 staining (a known cell stress marker; Tsujino *et al.,* 2000) and IENFs were analysed. Bortezomib‐treated animals showed no significant loss of axon myelination, compared to vehicle‐treated controls at day 24 (*n* = 5, vehicle 6.651 ± 0.66 axons mm^−2^, bortezomib 7.815 ± 1.06 axons mm^−2^). Additionally, no significant difference in the quantity of ATF3‐positive nuclei was found between bortezomib and vehicle‐treated animals at day 24 (*n* = 6, vehicle 0.405 ± 0.18% + ve nuclei, bortezomib 0.408 ± 0.11% + ve nuclei). Also, IENF analysis did not identify a significant difference in the number of nerve crossings in the hind paw skin of vehicle and bortezomib‐treated animals at day 24 (*n* = 6, vehicle 15.66 ± 1.48 IENF mm^−2^, bortezomib 17.35 ± 1.59 IENF mm^−2^).

## Discussion

Here, we report a comprehensively characterized, translational rat model of BIPN, utilizing established evoked pain measures and novel, ethological techniques. In conjunction with development of this novel model, we found that ethosuximide, an antiepileptic compound, successfully inhibited bortezomib‐induced mechanical hypersensitivity for up to 24 h after administration, suggesting its potential use as a positive control agent for testing novel analgesics. In addition, we also find that global inhibition of ROS, through administration of PBN, can curb bortezomib‐induced mechanical hypersensitivity for several hours. We have shown that a clear mechanical hypersensitivity develops following bortezomib administration, evident in a dose‐related manner. Further dosing regimens were not investigated in this study as 0.2 mg·kg^−1^ is equivalent to the standard patient dosing at 1.3 mg·m^−2^ (Argyriou *et al.,* 2014) and higher dosing has previously been shown not to be tolerated in rats (Cavaletti *et al.,* 2007).

Clinically, Velcade® is recommended for dosing at 1.3 mg·m^−2^ on days 1, 4, 8 and 11 in a 21 day cycle in the first instance (Moreau *et al.,* 2012). In patients, dosing with Velcade® has been shown to induce a 60% loss of platelets, which recovers over the 10 day rest period following dosing (Lonial *et al.,* 2005), and patients can develop thrombocytopenia and may require platelet transfusions. The schedule of dosing followed in our investigation was designed to mimic the intermittent dosing of one cycle of clinically administered Velcade®. Many existing animal models administer bortezomib at a dose higher than that recommended clinically and/or over a longer period, not reflecting the rest period included in the 21 day patient cycle. Additionally, dosing at 0.3 mg·m^−2^ i.v. was fatal to rats, and animals showed distress and decreased weight gain following 0.2 mg·m^−2^ i.v. administration (Cavaletti *et al.,* 2007). Thus, the pattern for one bortezomib administration cycle was also followed here to avoid animal health issues in accordance with the 3Rs principles. We did not dose i.v., or for longer periods, to minimize the impact on animal welfare, whilst reflecting the intermittent nature of systemic Velcade® administration in patients.

Existing models of BIPN have also reported mechanical hypersensitivity due to bortezomib dosing, but in a shorter‐lasting manner, 29 days (0.2 mg·kg^−1^ bortezomib; Yamamoto *et al.,* 2015) and 54 days (0.2 mg·kg^−1^ Velcade; Robinson *et al.,* 2014), compared to ~80 days in our investigation. Dosing with bortezomib has also previously been shown to induce cold allodynia at days 11 and 15 (Zheng *et al.,* 2012; Yamamoto *et al.,* 2015), whereas Velcade® administration was not associated with development of cold allodynia following four doses at 0.15 mg·kg^−1^ (Robinson *et al.,* 2014). In conjunction with long‐lasting mechanical hypersensitivity, we found evidence of a short‐lasting cold allodynia resulting from four doses of 0.2 mg·kg^−1^ Velcade®, evident at day 14 only. It is unlikely that long‐lasting bortezomib‐induced mechanical hypersensitivity and short‐term cold allodynia are due to impaired animal health or motor function as no weight difference or alteration in rota‐rod ability was evident between groups. Collectively, these data suggest that drug formulation, dose and dosing regimen are important factors in developing models of BIPN.

Patients report painful paraesthesia, hypoesthesia, cold allodynia, heat hypersensitivity and little motor impairment (Cata *et al.,* 2007; Argyriou *et al.,* 2008; Cavaletti and Jakubowiak, 2010), with a significant proportion of patients developing neuropathy after one treatment cycle (Richardson *et al.,* 2006; Broyl *et al.,* 2010). The model in this investigation closely mirrors the long‐lasting nature of bortezomib‐induced mechanical pain in patients. Heat hypersensitivity reported by patients (Cata *et al.,* 2007) has not yet been demonstrated in any rodent model of BIPN (see Hopkins *et al.,* 2016). We also did not observe heat hyperalgesia. One mouse study reported heat hypoalgesia, although this may be related to the high dosage used (Bruna *et al.,* 2010). Our investigation did not report any motor impairment following rota‐rod assessment, and it was also absent in another study which utilized Velcade® at a lower dose (0.15 mg·kg^−1^; Robinson *et al.,* 2014). These studies correlate with the lack of motor impairment observed in the human condition (Park *et al.,* 2013).

Clinically, Velcade® is administered intravenously or subcutaneously. Data have shown that subcutaneous administration results in an equivalent plasma level of bortezomib to intravenous administration. Additionally, both administration routes displayed comparable proteasome inhibition levels. The site of subcutaneous administration, thigh or abdomen, was also shown not to affect pharmacokinetic and pharmacodynamic parameters (Moreau *et al.,* 2011; Moreau *et al.,* 2012). There does not appear to be an agreed consensus regarding neuropathy incidence and administration routes, with studies reporting contrasting results (Moreau *et al.,* 2011; Arnulf *et al.,* 2012; Minarik *et al.,* 2015), suggesting that neuropathy prevalence is primarily linked to dosage. One animal study has administered Velcade® subcutaneously (Bruna *et al.,* 2010); however, this study had a high cumulative dose (12 mg·kg^−1^). The researchers state that this cumulative dose is equivalent to patients' receiving eight 21 day cycles of Velcade®; however, animals were dosed over 6 weeks without the rest period included in the 21 day patient cycle. This mouse model displays motor impairment, not been observed in patients, as well as reduced myelinated sciatic fibres and a loss of IENFs in bortezomib‐treated mice. The model characterized in this study did not find loss of motor function or reduced axon myelination and IENF crossings in bortezomib‐treated animals compared to vehicle‐treated controls. Previous studies have identified degeneration of myelinated sciatic nerve fibres following the same dosing regimen (albeit using non‐Velcade® bortezomib) used in this study, but an absence of ATF3‐positive staining (Yamamoto *et al.,* 2015). Another investigation has reported, but did not quantify, ATF3‐positive staining in the DRG following bortezomib administration following a significantly higher cumulative dose of 6.4 mg·kg^−1^ (Carozzi *et al.,* 2013). We did not observe any significant difference between the number of ATF3 positive nuclei in the DRG of bortezomib‐ and vehicle‐treated animals at the peak mechanical hypersensitivity time point in this study. Cavaletti *et al*. (2007) reported satellite cell vacuolization in schwann cells of the DRG and damaged mitochondria but only following cumulative dosing of 2.4 mg·kg^−1^, with a number of lower dosing regimens, akin to the cumulative dose used in this model, not causing these changes (Cavaletti *et al.,* 2007). Collectively, this suggests that neurodegeneration is associated with higher doses of bortezomib.

Our investigation did not find any change in burrowing activity in this model of bortezomib‐induced peripheral neuropathy, when examined during the injection period or on established mechanical hypersensitivity time points. Burrowing is an innate, spontaneous activity displayed by most rodents and is thought to be ethological, compared to standard behavioural methods which examine responses to evoked pain and thus reflect a better system to assess ongoing pain and potential analgesics. Burrowing has been shown to be sensitive to pharmacological treatments and induced conditions, including peripheral nerve injury, joint inflammation and antiretroviral‐associated neuropathy models (Deacon, 2006; 2009; Andrews *et al.,* 2012; Huang *et al.,* 2013; Rutten *et al.,* 2014; Muralidharan *et al.,* 2016; Wodarski *et al.,* 2016). This study found no difference in burrowing activity in bortezomib‐treated animals, compared to concurrent vehicle‐treated controls at any time point investigated. In contrast, paclitaxel administration impaired burrowing activity (Griffiths *et al*., submitted). Although a large, multicentre study has established a standard burrowing protocol for CFA‐induced inflammatory pain (Wodarski *et al.,* 2016), it may be that different pain models affect burrowing behaviour in a diverse manner.

Voluntary wheel running, particularly in a home cage or home cage‐like environment, provides a stress‐reduced method of testing in which the animal can be monitored objectively. Wheel running has been identified as a natural, elective behaviour (Meijer and Robbers, 2014) and can be assayed continuously or over a specific time period, for instance, following administration of a potential novel analgesic, without needing an experimenter to be present. We found that bortezomib‐treated animals, which showed mechanical hypersensitivity at the same time points, ran at greater distances when compared to concurrent vehicle‐treated animals over the time course examined. However, pairwise analysis did not identify any significant change in total distance travelled by bortezomib‐treated animals compared to concurrent vehicle‐treated controls. No other parameters (active time, access count, mean and maximum speed and maximum acceleration) showed any significant differences between groups, and time point analyses were all non‐significant. Wheel running has previously been shown to be sensitive to a number of pain models, including arthritis (Cobos *et al.,* 2012; Kandasamy *et al.,* 2016), osteoarthritis (Stevenson *et al.,* 2011) and paclitaxel‐induced peripheral neuropathy (Griffiths *et al*., submitted). The measures of spontaneous behaviour used in the characterization of this model of bortezomib‐induced peripheral neuropathy have not identified any significant deficits following administration of bortezomib. This is indicative of good health in the animals; voluntary wheel running was conducted during the night cycle, and burrowing was carried immediately prior to the dark cycle initiating. Rats are nocturnal and have been previously shown to be most active during the night cycle (Eikelboom and Mills, 1988); in addition, minor motor impairment has only been reported in a small number of patients receiving Velcade® (Argyriou *et al.,* 2008; Cavaletti and Jakubowiak, 2010). Therefore, the absence of impaired burrowing activity and voluntary wheel running behaviour may be an accurate representation of the lack of motor impairment reported by patients experiencing BIPN.

Currently, the only analgesic which is recommended for treating existing chemotherapy‐induced neuropathy, namely, oxaliplatin‐induced, is duloxetine (Hershman *et al.,* 2014; Majithia *et al.,* 2016). A very small study has shown duloxetine to be effective against BIPN in patients (Hirayama *et al.,* 2015); however, comprehensive clinical trials with duloxetine and bortezomib‐induced peripheral neuropathy have yet to be conducted. A number of existing analgesics have been tested in an animal model of bortezomib‐induced neuropathy (Yamamoto *et al.,* 2015) and have been shown to be effective in the short term, although the mechanical hypersensitivity described in this model was significantly shorter in duration than that described in this investigation, potentially not reflecting the patient experience of long‐term neuropathy following bortezomib administration. The model described by Yamamoto *et al*. (2015) showed that administration of tramadol, pregabalin, duloxetine and mexiletine could ameliorate bortezomib‐induced mechanical hypersensitivity, but only the effects of pregabalin exceeded 1 h (up to 3 h; Yamamoto *et al.,* 2015). In our investigation, administration of another calcium channel blocker, ethosuximide, was shown to robustly inhibit bortezomib‐induced mechanical hypersensitivity at 1 and 3 h after a single administration. Systemic administration of ethosuximide has previously been shown to induce a dose‐dependent reduction in mechanical hypersensitivity in a rat model of paclitaxel‐induced pain (Flatters and Bennett, 2004) and analgesic in rat models of traumatic nerve injury (Dogrul *et al.,* 2003; Hamidi *et al.,* 2012; Goyal *et al.,* 2015). There is also evidence of calcium dysregulation in bortezomib‐induced cell death, with bortezomib causing transient intracellular ER calcium release, resulting in mitochondrial‐calcium influx and caspase activation (Landowski *et al.,* 2005). This transient release may alter the membrane potential of neurons and induce spontaneous action potentials, propagating pain signalling associated with bortezomib administration. In conjunction with these data, Robinson *et al*. (2014) reported that four systemic administrations of 0.15 mg·kg^−1^ Velcade® induced increased firing and, long‐lasting after discharge, of spinal wide dynamic range neurons in rats with confirmed mechanical hypersensitivity (Robinson *et al.,* 2014). Although bortezomib is not known to cross the blood–brain barrier, these findings suggest that the effect of bortezomib administration on transient ER calcium release could involve in the spread of altered membrane potentials across the PNS to the CNS, leading to central sensitization as observed in other pain models (Ji *et al.,* 2003). Ethosuximide is a known T‐type calcium channel blocker; however, there is evidence that ethosuximide can also inhibit persistent sodium and calcium‐activated potassium currents (reviewed: Rogawski and Loscher, 2004). Therefore, the effective inhibition of bortezomib‐induced mechanical hypersensitivity by ethosuximide may not be through calcium channels alone. However, the potent action of ethosuximide against bortezomib‐induced mechanical hypersensitivity in our study supports a role of calcium dysregulation in the propagation of BIPN and emphasizes the potential for ethosuximide as a positive control for the testing of novel analgesics.

Bortezomib has also been shown to directly affect mitochondria, causing accumulation of ubiquitinated proteins within the mitochondrion, leading to a reduction in mitochondrial membrane potential and increased reactive oxygen production *in vitro* (Maharjan *et al.,* 2014). This provides evidence that *in vivo* ROS scavenging may be an effective target in understanding and preventing BIPN. Neuronally derived mitochondrial ROS have previously been shown to have a significant role in painful neuropathy associated with another chemotherapeutic, paclitaxel (Flatters and Bennett, 2006; Fidanboylu *et al.,* 2011; Griffiths and Flatters, 2015; Duggett *et al.,* 2016). In conjunction with these data, the global ROS scavenger, PBN, has been shown to ameliorate paclitaxel‐induced pain in the rat (Kim *et al.,* 2010; Fidanboylu *et al.,* 2011). Systemic administration of PBN at a time point where BIPN was established showed a significant reduction in mechanical hypersensitivity over 3 h after dosing. Taken together, the reduction of BIPN‐induced mechanical hypersensitivity following administration of a single dose of ethosuximide or PBN provides support for a role for calcium and ROS in the potentiation of BIPN.

In summary, this study describes a translational rat model of BIPN, induced by intermittent administration of clinical formulation Velcade®, to mimic the first bortezomib treatment cycle. This model displays long‐lasting dose‐dependent mechanical hypersensitivity and transient cold allodynia, with no motor deficits, reflecting symptoms typically reported by patients. No effects of repeated bortezomib administration were observed on novel ethological activity measures. In addition, we have identified ethosuximide and PBN as effective inhibitors of bortezomib‐induced mechanical hypersensitivity providing insight into the potential mechanism of action through which BIPN develops. Furthermore, these drugs could be used as positive controls in the assessment of novel analgesics in behavioural studies. Finally, ethosuximide is a well‐tolerated widely used treatment for absence epilepsy and therefore may have therapeutic potential for the treatment of bortezomib‐induced neuropathy in patients.

## Author contributions

S.J.L.F. conceived and designed the study. N.A.D. performed experiments and data analysis. N.A.D. and S.J.L.F. wrote the manuscript.

## Conflict of interest

The authors declare no conflicts of interest.

## Declaration of transparency and scientific rigour

This Declaration acknowledges that this paper adheres to the principles for transparent reporting and scientific rigour of preclinical research recommended by funding agencies, publishers and other organisations engaged with supporting research.
